# Coevolutionary attack and avoidance strategies in predators and grouping prey

**DOI:** 10.1098/rstb.2025.0202

**Published:** 2026-10-15

**Authors:** Daisy A. B. Cooper, James E. Herbert-Read

**Affiliations:** Department of Zoology, University of Cambridge, Cambridge, UK

**Keywords:** predation, arms race, predator–prey, hunting, social, group living, defence

## Abstract

The formation of groups by prey is one of the most widespread evolutionary responses to predation. However, while the anti-predation benefits provided to prey within groups have been extensively explored in the literature, the subsequent response of predators to grouping behaviour has lacked attention. In particular, what strategies predators use to attack grouping prey, and whether predation strategies have evolved to explicitly exploit the behaviour of prey groups, remains underexplored. In this review, we discuss the behavioural strategies predators have evolved to counteract the collective defences of prey groups, and in some cases capitalize on the prey's grouping behaviour. Specifically, we focus on the different strategies predators use to break apart and disrupt groups, while also exploring how predators capitalize on cohesion and coordinated movement to increase the likelihood of a successful attack. Overall, this review challenges the assumption that grouping is universally advantageous for prey, showing that predators have evolved a variety of successful strategies to overcome, and even exploit, grouping prey.

This article is part of the theme issue ‘Multi-predator feeding aggregations in pelagic waters’.

## 1. Introduction

Predators and their prey are engaged in coevolutionary arms races, whereby predators evolve adaptations to better exploit their prey, while prey evolve adaptations to reduce this exploitation. A well studied behavioural response of prey to predators is the evolution of grouping. Importantly, as in the arms races of other predator–prey interactions, how prey form groups and interact within them is a direct reflection of the strategies their predators use to hunt them. For example, because predators are more successful in attacking prey if they remain undetected [1], prey have evolved mechanisms to quickly acquire information gained about threats by others in the group [2,3]. As group size increases, many eyes result in an increase in the likelihood that predators are detected [4,5], and group vigilance, therefore, reflects the prey's objective to detect predators. Similarly, since some predators require selecting and ‘locking-on’ to a single prey during an approach, coordinated prey movement has been proposed as an adaptation to induce ‘confusion effects’, reducing the ability of predators to achieve this objective [6]. Additionally, because predators become satiated and often only target single prey items sequentially, as group size increases, each individual's risk of being predated is diluted among group members [7]. Prey grouping, therefore, often reflects a response to those predators that target individual prey or small portions of groups at a time. Ultimately, the strategies predators use to attack prey directly shape the social rules that prey have evolved to counteract such attacks, and therefore which anti-predator benefits of grouping are selected for [8].

While the anti-predator benefits of grouping have been extensively explored in the literature [9], the adaptations predators use to target individuals in groups have received less attention in these coevolutionary arms races. From the predator's perspective, as it becomes more difficult to attack individuals within groups, predators should evolve strategies to become more effective at isolating and targeting individual prey. In other cases, predators may evolve strategies that exploit prey grouping, improving capture success rates by capitalizing on how prey behave in groups. Indeed, there are natural enemies of a group-living species that exploit the social nature of groups, as is evident in many social parasites and pathogens where the risk of infection increases with host group size [10]. The strategies predators have evolved to capitalize on prey grouping, however, remain underdiscussed.

This review explores the strategies predators use to hunt grouping prey. Our focus is primarily on fission–fusion animal groups—those with fluid membership, minimal dominance hierarchies and open group boundaries (such as in pelagic waters; the focus of this special issue), although we include some examples of closed-entry social systems. We concentrate on the latter stages of the predation sequence, after groups of prey have been detected by predators. First, we focus on the behavioural strategies predators use to break down group defences, thereby attempting to erode the benefits prey achieve from grouping. We then discuss how some predators may attempt to exploit the grouping responses of prey, discussing how predators that attack intact groups of prey may capitalize on both the cohesion and the coordination of prey groups to increase their attack success (figure 1). Recognizing these predation strategies as only one side of this reciprocal evolutionary arms race, we further discuss how grouping prey may respond to predation strategies that successfully capitalize on prey grouping. Throughout, we focus on the behavioural strategies of predators and prey, but also include examples of morphological adaptations involved in targeting grouping prey. Many of the examples provided are taken directly from, or are applicable to, multi-predator feeding aggregations (MPFAs) in pelagic waters (the theme of this special issue). Overall, our aim is to highlight the interconnectedness of the strategies and counter-strategies that grouping prey and predators adopt for their defence and attack.

**Figure 1.**
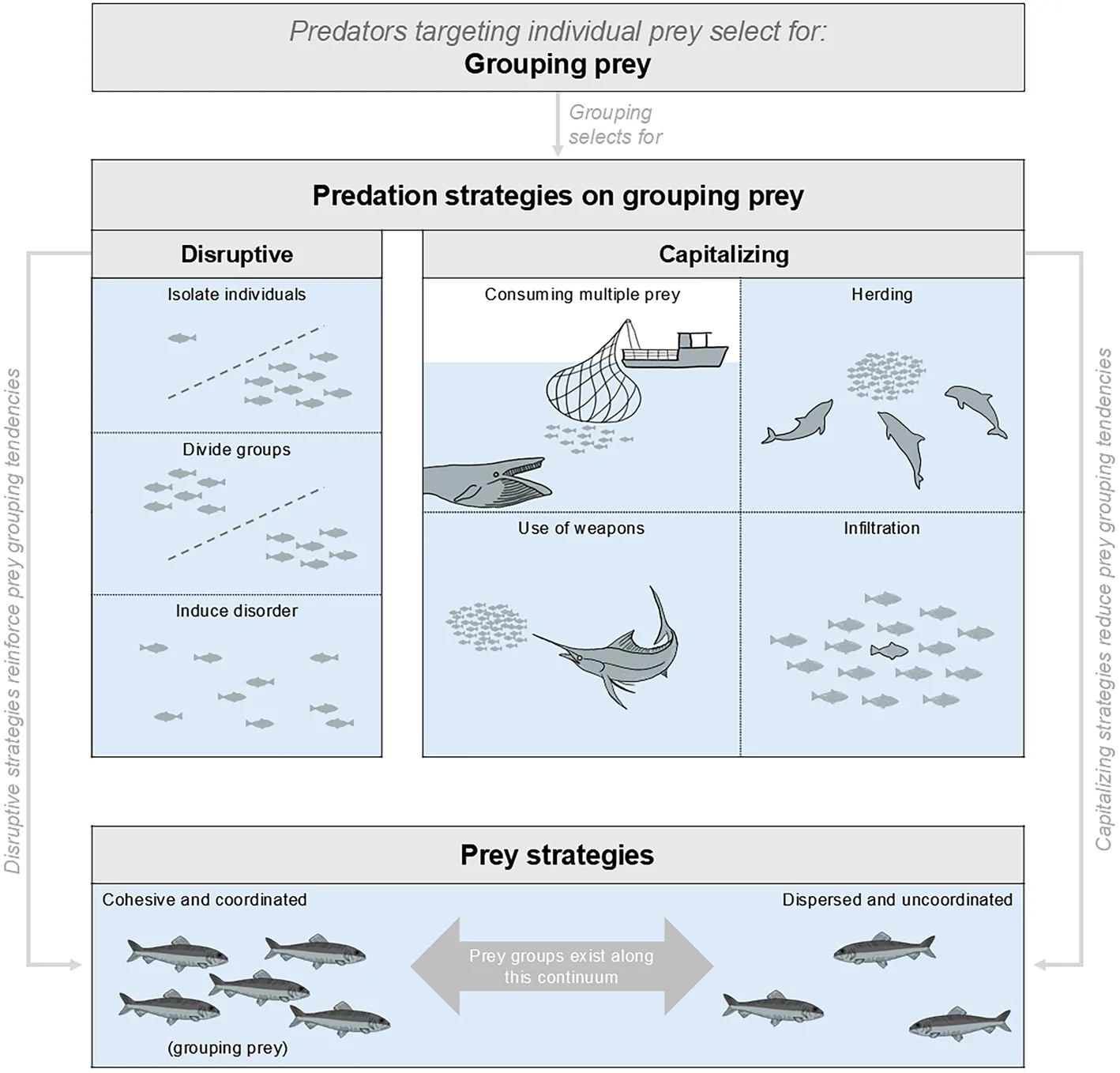
A schematic summarizing the content of the review, illustrating the strategies predators have evolved to attack groups of prey, highlighting potential evolutionary responses (grey arrows) of both prey groups and predators to counteract the strategies adopted by their enemies. Predators that target individual prey select for prey grouping, which in turn leads to predators adopting a variety of strategies to successfully hunt grouping prey. Two main classes of predation strategies are identified: those that disrupt group defences, and those that capitalize on the behaviour of grouping prey. These strategies in turn affect how prey behave in groups.

## 2. Predation strategies that break down group defences

### (a) Strategies to isolate individuals and divide groups

If attack success is reduced when prey form larger groups, predators should adopt strategies to mitigate these effects. In some cases, a reduction in attack success appears attributable to the ‘confusion effect’, where individual prey become more difficult to target when moving amongst other moving individuals. Predators, however, often show a tendency to preferentially target individual prey that differ phenotypically from the majority of group members [11–14], termed the ‘oddity effect’. It has been hypothesized that this allows predators to minimize the confusion effect, since an ‘odd’ individual appears more salient amongst other group members. Preferentially targeting ‘odd’ individuals, however, does not always reduce the time taken to successfully attack odd individuals compared with other prey, suggesting targeting phenotypically dissimilar prey does not eliminate the confusion effect [12]. Moreover, given that the oddity effect will select for homogeneous prey groups, predators require other strategies to successfully target individuals within groups.

One strategy predators can use to improve hunting success on grouping prey is to break apart, or reduce the size, of prey groups. By isolating individuals, or a subset of individuals, from the group, these prey no longer retain the anti-predation benefits of grouping, and predators therefore can achieve increased attack success by targeting these individuals. A simple, yet effective, strategy to do this is to perform repeated attacks on prey groups. Theoretical models of solitary avian predators attacking flocks of prey suggest that repeated attacks in quick succession are predicted to achieve a high attack success through preventing prey from re-forming, and allowing predators to get closer to their prey [15]. Indeed, multiple attacks by European sparrowhawks (*Accipiter nisus*) on groups of redshank (*Tringa totanus*) resulted in a reduction in flock size. As a result, the predicted attack success of sparrowhawks increased by as much as 1.6-fold as group size decreased, potentially because these smaller groups benefited less from confusion effects and were slower at detecting approaching predators [16].

Pursuit predators also attempt to isolate individuals by separating individuals from the group during the chase. At faster speeds, smaller directional errors become magnified, resulting in an increased likelihood of prey becoming isolated from the group [17]. Indeed, predatory striped marlin (*Kajikia audax*) have higher success at targeting mobile prey compared with stationary groups because there is an increase in the number of prey isolation events during chases [17]. Modelling suggests that pursuit predation strategies that result in the dispersal or isolation of individuals from prey groups can be especially beneficial to predators when the intact prey group induces confusion effects [18]. Predators can also use their superior stamina to attack prey that choose to flee as a defence strategy. This can facilitate the preferential targeting of weakened or injured individuals, which may be easier to attack once separated from the group, as prey groups tend to self-assort based on physical ability during a chase [19,20]. Slower individuals, which are unable to keep pace with the group, become isolated and therefore more vulnerable to attack [21]. Pursuit predators, such as Alaskan wolves (*Canis lupus*) and African wild dogs (*Lycaon pictus*), will chase prey groups until individuals that are typically weaker owing to age, illness or injury become separated from the group [21–23]. Indeed, impalas (*Aepycerus melampus*) that were successfully captured by African wild dogs (*Lycaon pictus*) typically showed lower marrow fat levels (used as a proxy for health) than the population average [20]. Some predators also use weapons to inflict injuries on prey within groups, increasing the number of weakened prey within groups. Sailfish (*Istiophorus platypterus*), for example, injure sardines (*Sardinella aurita*) within schools with ‘taps’ and ‘slashes’ from their microtooth-covered rostrum, and these injured individuals, unable to match the speed of their conspecifics, often become separated from the group and are subsequently captured [19].

In other cases, rather than isolating individuals, attacks by predators can split off smaller groups of prey from the main herd, with predators subsequently focusing attacks on these smaller groups. Groups of orca (*Orcinus orca*) split large aggregations of herring (*Clupea harengus*) into small, dense schools to attack them [24]. Groups of predatory yellowtails (*Seriola lalandei*) form coordinated lines that split up large schools of both jack mackerel (*Trachurus symmetricus*) and grunt (*Lythrulon flaviguttatum*) into smaller schools [25]. Similarly, sailfish (*Istiophorus platypterus*) break off smaller groups of their prey (*Sardinella aurita*) from larger groups containing thousands of fish, and herd these smaller groups to the surface [26]. It may be more efficient, in terms of prey caught per unit time, to portion off a part of a group and concentrate attacks on this fragment before portioning off another fragment. While not explored, models equivalent to the marginal value theorem [27] could be repurposed to explore the conditions where such a strategy benefits individual predators.

### (b) Attacking to disrupt group structure

Many of the anti-predation benefits of grouping are dependent not only on the group's size but on its structure. For example, the effectiveness of the confusion effect is thought to depend not only on group size and visual homogeneity of members, but on group density and organization [6,28]. Fish schools have also been proposed to adopt densities to ensure information about an attack can propagate between group members more effectively [29]. Similarly, scale-free correlations in starling flocks have been suggested to allow information from informed group members to propagate across entire groups [30]. Importantly, relatively minor deviations in individual prey behaviour and group organization can have disproportionately large effects on these anti-predation benefits of grouping. For example, increased variance in individuals' speeds in starling flocks can cause scale correlations to decay more rapidly with distance, limiting information spread to only nearby neighbours [30]. Similarly, relatively minor changes to group density can impede information from being propagated between group members [29]. If predators can induce changes in group structure, therefore, they have the opportunity to counteract the benefits of grouping, potentially increasing their attack success.

One way predators can disrupt the organization of a prey group and maintain it in a disordered state is by using successive attacks on prey groups. Groups of predatory sea trout (*Cynoscion nebulosus*) attack schools of juvenile Gulf menhaden (*Brevoortia patronus*) in formation by forming lines of up to five individual predators that manoeuvre through the school. This strategy, as well as being more likely to fragment prey groups (see above), is associated with a decrease in the density of prey groups. By disrupting school structure, prey are expected to receive an increased predation risk, owing to the predators being able to get closer to individual prey and information transfer being impaired in disorganized groups [31]. Flatheaded gudgeon (*Philypnodon grandiceps*) perform repeated attacks on groups of Pacific blue-eyes (*Pseudomugil signifer*) and appear to preferentially target groups when they are in configurations they do not usually adopt [32]. A similar phenomenon is observed in black-headed gulls (*Larus ridibundus*) attacking schooling bleak (*Alburnus alburnus*). Instead of a single predator attacking multiple times, here, increased numbers of gulls attacking impacted the prey school's structure. Gulls attacking in larger groups also achieve a higher success rate, potentially owing to the increased vulnerability of the fish in these more dispersed groups [33]. Overall, this suggests that predators may increase their attack success by disrupting the cohesion of prey groups through performing multiple attacks.

Predators can also induce disorder in prey groups by forcing groups to split, with rejoining events creating momentary disorder when the sub-groups reunite. For example, schools of sandeels (*Ammodytes* sp.*)* kept in tanks with predatory mackerel (*Scomber scombrus*) were occasionally observed to split into two sub-groups, with these splits occurring most commonly when the school was trapped between two or more mackerel, or against the wall of the tank. When these sub-groups rejoined into a single larger group, leaders of each sub-group aligned and swam together in the same direction, while individuals towards the back were more likely to remain unpolarized during the merging process. The authors termed this disorder at the back of the newly re-formed group a ‘confusion zone’ [34], and suggested that predators may achieve increased attack success by targeting individuals within it. This prediction is supported by observations of wild predators frequently attacking prey groups as they merge [35], though whether these attacks are preferentially directed at the confusion zone specifically remains to be established. In the open ocean, where physical boundaries are absent, multiple predators hunting together may be especially successful at inducing splits, thereby allowing individuals to exploit the disorder that is created when fragmented sub-groups reunite.

Pressure to disrupt group organization may have also driven an increase in association between multi-species predators that hunt in groups. While there is evidence that, in the presence of multiple predator species, feeding rates can be reduced owing to competitive interference [36,37] or are unaffected by the presence of other predators altogether [38], in some cases multi-species predators may benefit from hunting together [39]. Moreover, while some predators (e.g. aerial predators) are likely to disproportionately benefit from attacking with other predators [40], if disorganizing prey groups increases capture success, all predators within this multi-species predator aggregation may benefit to some degree from increased foraging efficiency as a result of this interspecific feeding strategy. This could explain why multi-species aggregations, specifically between seabirds (e.g. terns, gannets and gulls) and large subsurface predators (e.g. sailfish, delphinids and tuna), commonly hunt together. Rather than these multi-species predators simply aggregating on patchy resources, there may be benefits for both aerial and aquatic predators attacking prey groups simultaneously. Indeed, models of multi-species predators attacking prey groups find that the spatial disorder of the prey group is kept highest (and prey group density lowest) when the frequency of attacks prevents the group from returning to an organized state between attacks [41]. Moreover, prey groups are theoretically kept in a disorganized state for longer if there is a mixture of aerial and underwater attacks by different predatory species [41]. These predictions are supported by empirical observations. Terns (*Onychoprion* sp.) attack schools of anchovies (*Encrasicholina* sp.) and sardinellas (*Amblygaster* sp.) alongside Indo-Pacific sailfish (*Istiophorus platypterus*). Here, terns preferentially attack schools during periods of low cohesion following attacks by sailfish [40]. Additionally, Cape gannets (*Morus capensis*) feed on groups of sardines (*Sardina pilchardus*), alongside other aquatic predators (commonly the long-beaked common dolphins *Delphinus capensis*), with the foraging success of gannets being highest when there are preceding attacks by other predators in the moments before their attack [39]. The mechanism by which feeding rates are thought to be improved when multi-species predators hunt together is through prey having conflicting predator-specific defences, which increase their vulnerability to the other predator species present [42].

## 3. Predation strategies that capitalize on prey grouping

While above we have provided examples of strategies that are used to break up or otherwise manipulate the structure of prey groups, in other cases predators are often observed attacking groups of prey without attempting to disrupt the group. In some cases, this may be due to predators being unable to disrupt prey defences, but alternatively, some predators may have evolved predation strategies that allow them to actively capitalize on the prey's grouping behaviour. In turn, these strategies may allow predators to increase their hunting success on grouping prey.

### (a) Capitalizing on social cohesion

While grouping may reduce individual risk through the dilution effect when predators target individual prey [7,43], some predators consume multiple prey in a single feeding event. Moreover, by aggregating together, small prey that are unprofitable for large predators at lower densities [44] become an energetically viable food source when at higher densities [45]. To exploit these prey groups, some predators have developed specialized behaviours or morphologies to capture large numbers of small prey simultaneously. Several large terrestrial predators, for example, have evolved to exploit grouping invertebrates such as ants and termites [44], with giant anteaters, aardvarks and echidnas evolving specialized morphological features, such as long, adhesive tongues and elongated snouts, to exploit the high local density of prey [46–48]. Similarly, chimpanzees (*Pan troglodytes*) use tools to consume multiple prey simultaneously and thereby increase feeding rates [49].

In aquatic environments, gape-limited predators can drive prey to form groups, which non-gape-limited predators can capitalize on by consuming multiple prey simultaneously (see figure 1). Blue whales (*Balaenoptera musculus*), for example, use bulk filter feeding, engulfing large volumes of seawater and filtering out krill and small fish through their baleen plates, and consuming large portions of the prey group. This feeding strategy can provide blue whales with energetic efficiencies that are approximately an order of magnitude larger than other marine mammals [45]. This bulk feeding strategy, however, is only energetically beneficial when prey density reaches a critical value (0.1 kg m^–3^), below which a net loss of energy is predicted [45]. Bulk feeding, therefore, not only capitalizes on, but is dependent on, prey grouping. Other predators have similarly evolved morphological adaptations to exploit high prey densities. Crab-eater seals (*Lobodon carcinophaga*) filter out small aquatic prey using their multi-cusped teeth [50], and flamingos (*Phoenicopterus* spp.) similarly use the lamellar meshes in their L-shaped beaks to extract suspended food particles, including algae and small crustaceans, from the water [51]. Such adaptations are analogous to the methods used by commercial fisheries, such as purse seining, exploiting fish that are aggregated in dense schools [52]. Ultimately, social attraction of prey species can create opportunities for predators to consume large portions of the group, allowing them to take advantage of condensed resources.

Predators living in environments with low prey densities may also be able to exploit the grouping responses of prey to force dispersed prey to aggregate, thereby creating locally rich patches of resources. Indeed, groups of predators herding dispersed prey into dense aggregations is a strategy used by a variety of pelagic aquatic predators [24,53–56], and is dependent on the prey's social attraction to others. Spinner dolphins (*Stenella longirostris*), for example, feed on fish and must consume approximately 1.25 prey items per minute during foraging to meet their daily energetic requirements [57]. To achieve this, spinners hunt in groups, progressing through a series of coordinated stages to isolate and encircle patches of prey. Once prey density peaks, which can be up to 200 times above the mean background density, the dolphins start to feed [58,59]. This manipulated increase in prey density, which appears to rely on the coordinated action of multiple individuals, not only provides a concentrated energy source but is also thought to constrain the manoeuvrability of the prey and suppress their ability to avoid attacks [59].

Exceptionally high prey densities can further allow predators' weapons to be used more effectively compared with low prey density conditions. While the role of weapons is primarily to allow larger predators, which themselves are less agile [60], to more closely match the superior manoeuvrability of their prey, under high prey densities these weapons can facilitate novel predation strategies. Thresher sharks (*Alopias pelagicus*), for example, use powerful tail-slaps to stun prey that form dense schools. This strategy, in combination with the high density of their prey, is thought to allow sharks to capture multiple individuals simultaneously [61]. Similarly, orcas (*Orcinus orca*) have been observed using tail-slaps on prey aggregations they have herded into artificially high densities, and these strikes may impact as many as 33 fish in a single pass. This strategy is thought to overcome the confusion effects that affect predators targeting individual prey [62]. Sailfish (*Istiophorus platypterus*) employ another tactic, slashing their bills to injure several fish within tightly packed bait balls. This technique capitalizes on the close proximity of prey to facilitate injuring multiple prey at once [19,26,63].

The formation of dense prey groups may also counterintuitively play a role in allowing predators to successfully target individual prey in groups. While the confusion effect is commonly thought to reduce individual risk as prey become more difficult to isolate in prey groups, some predators may adopt strategies to capture individual prey that would be ineffective under low prey density conditions. For example, when Swainson's hawks (*Buteo swainsoni*) attack swarming Mexican free-tailed bats (*Tadarida brasiliensis*), instead of ‘locking-on’ to a single target to follow, they use a constant bearing angle to turn towards a fixed point within the swarm, attacking whichever prey the hawk collides with [64]. By adopting a constant turning angle, the prey that is targeted appears stationary to the predator, thereby reducing potential visual confusion effects [64]. By decoupling target selection and steering in this way, the predators achieve equal attack success rates when attacking bats inside and outside of the swarm [64,65]. This tactic is likely to be less successful when attacking sparser groups where collisions are less likely.

### (b) Capitalizing on coordination

Individuals in prey groups appear to use simple behavioural rules to coordinate their movements. For example, the coordinated movement of groups can be explained through simple rules that determine how individuals respond to their near neighbours' movements and positions [66–68]. Individuals may also follow simple rules that determine how they respond to an approaching predator, such as changing direction and increasing speed upon detecting a threat [69]. Within a coordinated group, therefore, these responses become somewhat predictable, because an individual's responses can be predicted by simple, reflexive rules to escape, but also because social interactions will constrain individuals in their movements [70,71]. It is this predictability that predators may exploit.

By anticipating the likely escape trajectories of prey, predators may be able to manipulate the movement of prey groups and herd them to locations where attacks are more successful. ‘Bison jumps’, for example, were used for over 5000 years by Prehistoric hunters in the North American Great Plains, whereby an understanding of how moving bison groups would respond to external stimuli allowed hunters to guide entire herds to hidden cliffs [72]. Indeed, many predators exploit the prey's tendency to move as a coordinated group and their instinct to flee from threats to control the movement of large groups. For example, many species of marine predators, such as dolphins [56,73,74], orcas [62,75], humpback whales [76] and teleosts [73,77], will drive large groups of prey to the surface of the water, to shallower regions, or even onto the shore. American white pelicans (*Pelecanus erythrorhynchos*) are limited to foraging in the upper 1.25 m of water owing to their neck and bill length. To overcome this constraint, groups of pelicans can swim together, using their bodies to drive groups of fish towards shallow water near the shore. This herding leads to an increase in capture rate for individual pelicans [78], with their gular pouches further allowing the capture of multiple fish at once. Groups of predatory yellowtails (*Seriola lalandei*) will similarly herd jack mackerel (*Trachurus symmetricus*) towards subtidal boundaries, where they then concentrate attacks [25]. By driving prey groups to boundaries, predators likely benefit by restricting the escape options of prey and potentially allowing predators to get closer to individuals during attacks. Importantly, very simple heuristic rules can produce effective herding and containment behaviour [79], and therefore predators may not require sophisticated coordination rules to achieve such goals.

In some cases, predators do not aim to herd prey towards boundaries, but rather keep the prey in one location where they can then focus attacks. This relies on predators restricting the multiple potential escape options of prey. Groups of predators, for example sailfish, appear to achieve this by surrounding the perimeter of the prey group, with individual predators then alternating attacks on the prey group [26]. In other cases, predators have developed novel strategies to contain prey, such as bubble-netting, in which predators, such as humpback whales (*Megaptera novaeangliae*), confine the prey group within a bubble ‘net’ before attack [80]. Similarly, bottlenose dolphins (*Tursiops* spp.) use a mud-ring hunting strategy, which involves stirring up the sediment around a prey group and then capturing fish as they jump from the water to escape [81]. Across these herding strategies, it is the prey's tendencies to remain cohesive that allow such predators to constrain and manipulate their prey's movements.

Predators may also capitalize on the coordination rules of their prey by attacking groups in ways that produce predictable escape responses that predators can exploit. For instance, predators are often observed attacking from the side or back of prey groups, and it has been suggested that this is not only because prey are more likely to be fleeing predators, but because attacks are more successful if the group is approached from specific directions. Striped marlin (*Kajikia audax*), for example, routinely attack schools of sardine prey (*Sardinops sagax caerulea*) from the side or from behind. Observations of attacks in the wild show that the majority of marlin attacks result in the prey group performing a ‘fountain effect’, a collective response where the prey group splits in two, with each sub-group arching around the predator before rejoining. These escape patterns emerge from a combination of individual escape angles with social interaction rules. However, fountain effects take longer to complete, and groups take longer to rejoin, if predators attack from the back of the schools. This suggests marlin may select to attack from certain angles to increase the likelihood of the group splitting [82]. Such predictability in prey escape behaviour given prey's coordination rules, therefore, may be exploited by predators to disrupt or fragment prey groups.

### (c) Infiltration of the group

The mechanisms by which prey in coordinated groups interact may also increase their vulnerability to infiltration by predators. Visual information is key for many animals to coordinate their grouping behaviour [83]. However, because the behavioural responses to neighbours must be rapid, it is unlikely that individuals attend to and extract all visual information about conspecifics before responding to them. Instead, they likely rely on simple visual heuristics [84], selectively respond to only a limited number of near neighbours [85], or may extract only simplified visual information to guide their behaviour [86]. Such mental shortcuts likely enable them to extract relevant positional and directional information, but this visual simplification may make prey susceptible to misdetecting concealed objects, or infiltration by individuals that are sufficiently similar in both appearance and behaviour. Some predators appear to exploit this vulnerability by mimicking their prey, infiltrating the group and avoiding detection.

When sailfish approach prey schools, they reduce their swimming speed to match that of their prey. As they insert their bill into the prey school, sardines do not appear to react to the presence of the bill [19], suggesting that the bill functions as a stealthy weapon to target grouping prey. The lack of response by the sardines may be influenced by the prey's schooling behaviour if schooling with others reduces their capacity to detect such weapons. Many marine predators have evolved similar, convergent morphologies characterized by pointed snouts and thin, elongated bodies. These shared features may all play a similar role in facilitating infiltration by delaying prey's escape response, as seen in taxa such as needlefish (Belonidae spp.), barracudas (Sphyraenidae spp.) and cornetfish (Fistulariidae spp.) [87].

Other predators use stealthy behavioural strategies to infiltrate prey groups without detection. Some micro-predators, which impose sub-lethal costs such as removal of skin, scales or fins, exploit the grouping behaviour of their prey in this way [88]. For example, the scale-eating tetra *Probolodus heterostomus* mimics and micro-predates upon *Astyanax fasciatus*, with observations in the wild showing that these two species commonly school together [89]. Similarly, the scale-eating cichlid, *Corematodus shiranus*, schools while eating scales from *Tilapia*, with which it has a strong superficial resemblance [90]. The success of these micro-predators is potentially facilitated by the social behaviour of their prey. In particular, if simplified visual heuristics used during schooling impair the prey's ability to distinguish between conspecifics and mimetic predators, this could facilitate micro-predators remaining undetected in the group. This hypothesis is supported by the finding that, in the absence of schooling, prey are able to identify and avoid their micro-predatory mimics [89].

An alternative, but not mutually exclusive, explanation for successful predator infiltration is that predators exploit the costs associated with prey abandoning grouping. Prey may incur sub-lethal costs by remaining in an infiltrated group, such as in groups with micro-predators, but these costs may still be lower than the risk of lethal predation faced by individuals that leave the group. In the case of the sailfish attacking sardines, the increased predation risk for a sardine leaving the group is generated by the same predators that are imposing the sub-lethal injury costs of remaining in the group [26]. For scale-eaters, the presence of other predators in the environment may deter prey from abandoning the invaded group. Some predators, therefore, may rely on the costs to prey of remaining within the group being lower than the expected cost of leaving, allowing predators to remain with and exploit concentrated patches of resources. This is supported by observations of prey schools invaded by scale-eaters, where prey groups only break apart when the ratio of scale-eaters to prey, and therefore the frequency of attacks, becomes higher [89].

Predators that infiltrate prey groups may also stand to gain more than simply increased access to prey. Solitary midges (*Aphidoletes aphidimyza*) infiltrate aphid colonies to feed on them. When in the colony, midges receive the additional benefit of a dilution effect, reducing their own risk of predation from predators that target them as well as the aphids [91,92]. Similarly, fish micro-predators resemble their mimicked prey in size [88], and therefore likely share the same predators. Infiltrating a prey group, therefore, may also benefit predators through mitigating their own predation risks. Ultimately, predation strategies that involve infiltrating prey groups may capitalize on multiple aspects of prey grouping.

### (d) Capitalizing on other anti-predation defences

Predators can exploit other behavioural defences of the prey group, such as mobbing behaviour, though this occurs within certain theoretical constraints. Mobbing occurs when prey, either alone or in groups, attack or otherwise harass a predator to deter it and reduce their risk of predation [93]. However, this strategy comes with a small, but not insignificant, risk of injury or predation, because it requires the prey to make themselves more visible and approach the predator [93]. Some predators appear to take advantage of this vulnerability by deliberately provoking mobbing behaviour as a hunting tactic. For example, slaty-backed forest-falcons (*Micrastur mirandollei*) have been observed to trigger mobbing in their prey, and target individuals that expose themselves in the process [94]. However, if predators exploit prey behaviours that have evolved to serve an anti-predation purpose, such predation strategies must remain rare; otherwise, natural selection would favour the loss of such behaviours. Indeed, observations by Smith [94] identified *Micrastur mirandollei* as the rarer of the two falcon species present, with the other falcon, *Micrastur rujicollis*, appearing to not induce mobbing as a predation strategy. The same theoretical constraint applies to the persistence of predation strategies that exploit other anti-predation defences of prey, such as grouping. To persist, these strategies must remain rare; otherwise they would select against the same behaviours that they capitalize on.

## 4. Responses by prey to predation strategies that capitalize on grouping

Recognizing that some predation strategies have evolved to capitalize on the behaviour of grouping prey, it is important to consider how prey have responded in this ongoing arms race. One potential strategy to impede predators from capitalizing on grouping is for individuals to depart from groups when faced with predators that adopt capitalizing strategies. However, such short-term dissolution is likely to be maladaptive in many cases. First, leaving a group may increase predation risk via oddity or marginal predation effects, where isolated individuals are disproportionately targeted by predators [95–97]. The fitness costs of departing from the group, therefore, are likely to be even larger than the costs of remaining with the group in these circumstances. This may lock prey into a ‘cruel bind’, where individuals tolerate the risks of belonging to the group because the alternative, solitary exposure, carries even greater risks.

If, however, prey can dissolve the group in a coordinated way, then individuals may swamp predators targeting isolated prey, with individuals potentially standing to benefit from a rapid group fission event. Such rapid, simultaneous, short-term decreases in social attraction triggered by immediate predation threat are observed in nature. ‘Flash expansions’ occur when individual prey move away from the centre of the group preceding a predation attack [34,98]. These responses are seen in many groups, including fish schools [99], insect swarms [98] and bird flocks [100]. For example, groups of anchovies (*Engraulis mordax*) perform flash expansions when their capitalizing predator, humpback whales (*Megaptera novaeangliae*), open their mouths to engulf shoals. In response, however, whales delay the time when they open their mouths, resulting in a large proportion of the school being caught despite such evasive manoeuvres [101]. Further evidence that prey groups disband under conditions where predators stand to gain from prey grouping includes the common finding that fish schooling is largely a diurnal phenomenon, with groups dispersing at night [102–107]. At night, the benefits provided by group-level visual defences, such as the confusion effect, may become redundant, and nocturnal predators may even rely on concentrated olfactory cues to detect prey groups [108]. In some cases, therefore, individuals can abandon grouping when these costs become excessive.

On an evolutionary timescale, it may not benefit prey groups to dissolve, even in environments with capitalizing predators. Prey may benefit from two key mechanisms of attack abatement: the dilution effect, where an individual's risk of being targeted decreases as group size increases, and the avoidance effect, where larger groups are less likely to be found by predators since finite populations mean larger groups are fewer and further away from predators [109,110]. Since the overall risk to individual prey is a combination of their probability of being detected and their probability of being attacked (i.e. attack abatement), prey may still benefit from grouping in the presence of capitalizing predators if grouping sufficiently decreases the likelihood of being detected to offset the increased risk of being targeted post-detection. Additionally, because environments are likely to contain multiple predators using different hunting strategies, the risks imposed by non-capitalizing predators would sustain the evolutionary pressure driving grouping, even in the presence of a low frequency of predators capitalizing on grouping behaviour.

Only under conditions where prey experience higher rates of mortality when grouping versus not, with mortality being a combination of detection probability and attack probability post-detection, can selection drive a shift away from grouping to solitary behaviour. Industrial fishing represents an extreme form of capitalizing predation, with ever-improving technology allowing the targeting and capturing of entire schools. Indeed, mass-capture fishing techniques are likely to be the most prevalent (in terms of biomass captured) capitalizing predators in the marine environment [111]. It has been suggested that industrial fishing could drive a decrease in group formation through selection against schooling behaviour [112–114], and experimental evidence demonstrates that larger schools of fish are more vulnerable to mortality from human fishing techniques [115]. Subsequent modelling has predicted that, as the prevalence of mortality by these mass-capture (capitalizing) fishing techniques increases in relation to natural predation pressure, individual prey should prefer smaller groups, resulting in a reduction in school sizes [116]. This may drive a shift away from group living in some marine systems.

## 5. Conclusion

While the evolution of prey grouping as an anti-predator defence has been well studied, comparatively little attention has been given to the adaptive responses of predators that attack grouping prey. This review has explored how predators have either developed strategies to break down or disrupt prey groups, preventing prey from benefitting from their group defences, or capitalized on prey grouping itself, exploiting behaviour of individuals in groups to their advantage. Strategies have evolved, including splitting prey groups, successively attacking with others to disrupt groups, bulk feeding, the use of weapons, herding and infiltrating the group. On an evolutionary timescale, while these strategies can improve the capture rates of predators targeting grouping prey, we have outlined reasons why such strategies are unlikely to select for an abandonment of grouping.

A recurring characteristic of hunts on aggregated prey is the simultaneous presence of both interspecific and intraspecific predators during feeding. The evolution of multiple predator feeding aggregations (MPFAs) has been suggested as an evolutionary response of predators to achieve increased attack efficiency on prey groups [117]. However, little attention has been given to the potential mechanisms underlying how MPFAs could result in increased feeding efficiency for individuals. Here, we discuss how multiple predators can manipulate prey groups, for example by increasing prey density or disorder, to enhance foraging success. By hunting together, feeding rates may be higher in MPFAs for some predators, as processes like herding, exhausting or injuring prey may be more successful when acting together. In other cases, the strategies some predators use to manipulate prey groups may advantage some predators more than others. For example, seabirds are thought to exploit prey that have been driven to the surface by aquatic predators [73,118], while baleen whales such as humpback whales (*Megaptera novaeangliae*) and fin whales (*Balaenoptera physalus*) have been observed feeding on bait-balls of prey formed by other predators, such as bottlenose dolphins (*Tursiops aduncus*) [119] and orcas [120]. Indeed, observations of interspecific MPFAs may be driven, in part, by the benefits some species gain from hunting with other species. Overall, while the specific mechanisms that result in some predators benefitting from MPFAs will likely vary between species, they are underpinned by the common goal of manipulating the behaviour of grouping prey.

New technologies now make it possible to more quantitatively assess the strategies predators use to hunt groups of prey. Drone technology, for example, has been particularly effective in allowing quantification of how marlin attack schools of sardines in MPFAs [17,40,82,121,122]. Given that experimentally testing the function of particular attack strategies on groups of prey is challenging under real-world conditions, like in the open ocean, these data can better inform theoretical models that can explore the function of such behaviour (e.g. [82]). Where experimental manipulations are possible under laboratory conditions, closed-loop robotic prey and predators could allow the dynamic attack rules of predators and prey to be decoded. Similarly, augmented reality environments where the properties of prey groups can be easily manipulated allow functional tests of different attack strategies. While previous explorations have provided valuable qualitative information on predation strategies [123–125], the tools now available for researchers allow the fine-scale decisions of predators based on the sensory inputs generated by prey groups to be decoded. This will be especially valuable when decoding the final stages of an attack sequence, where fine-scale adjustments to movements may become increasingly important for success [18,60,126,127]. Overall, such approaches will move the field from the largely qualitative approaches used to date, to more quantitative explorations that link a mechanistic understanding of the attack decisions by predators on grouping prey to their functional consequences. This will also enable a more rigorous comparison of the evolutionary and ecological drivers of predation strategies on grouping prey across taxa.

This review has also highlighted the need to challenge the view that prey grouping is universally beneficial to prey from an anti-predation perspective. Instead, prey grouping evolved in response to specific predation strategies, as one counter-move within a broader, cyclical evolutionary arms race [128]. Prey grouping has created an evolutionary challenge for their predators, and, in turn, some predators have evolved strategies that allow them to exploit multiple aspects of prey grouping, rather than being constrained by it.

## Data Availability

This article has no additional data.

## Theme

One contribution of 14 to a theme issue ‘Multi-predator feeding aggregations in pelagic waters’.
